# Practical Fraud Detection and Prevention in Incentivized Online Surveys: Secondary Analysis of the ADOPT Study

**DOI:** 10.2196/90159

**Published:** 2026-07-31

**Authors:** Douglas R Oyler, Sophia J Edgecombe, Emily A Babusci, Jennifer Dolly Prothro, Aaron L Begley, Marcia V Rojas-Ramirez

**Affiliations:** 1 Department of Pharmacy Practice and Science, College of Pharmacy, University of Kentucky, 760 Press Avenue, Lexington, KY, 40508, United States, 1 859-562-3038; 2 Ernest Mario College of Pharmacy, Rutgers, The State University of New Jersey, New Brunswick, NJ, United States; 3 Department of Oral Diagnosis, Medicine, and Radiology, College of Dentistry, University of Kentucky, Lexington, KY, United States

**Keywords:** data collection, fraud, surveys and questionnaires, patient reported outcome measures, oral surgical procedures

## Abstract

**Background:**

Digital surveys are increasingly integrated into clinical and public health research to capture patient-reported outcomes. However, concerns about fraudulent or duplicate responses threaten data integrity. Most of the literature on survey fraud focuses on open-access online recruitment, where bot-generated or anonymous entries are common, but far less is known about fraud patterns in clinic-linked, incentive-based surveys. Evaluations of the real-world implementation of fraud-deterrence strategies remain limited.

**Objective:**

This study evaluated whether implementing enhanced fraud-deterrence procedures in an incentive-based, clinic-linked, postprocedure survey reduced the prevalence of potentially fraudulent responses. Second, the study evaluated which indicators were most frequently triggered before and after implementation.

**Methods:**

This evaluation was conducted within the ADOPT (Alternatives to Dental Opioid Prescribing After Tooth Extraction) study. Eligible patients aged 12 to 25 years were recruited through QR-coded, clinic-distributed flyers and invitation cards. Participants completed a screening survey followed by an incentivized postprocedure survey between days 6 and 10 after tooth extraction. A midstudy protocol modification introduced enhanced fraud-deterrence measures in the screening process, including a phone number requirement, prohibition of email invitations, date of birth confirmation, and the use of a participant list with SMS text message invitations. For analysis, survey responses were categorized as “control” (before modification) or “intervention” (after modification). A 6-item scoring system assessing completion time outliers, submission time, repeated screeners, duplicated phone numbers, a blank recruitment source, and illogical response patterns was used to classify responses as potentially fraudulent (≥2 indicators). Sensitivity analyses evaluated thresholds from 1 to 3 indicators.

**Results:**

A total of 573 survey responses were included, with 122 in the control cohort and 451 in the intervention cohort. The overall prevalence of potentially fraudulent responses (50/573, 8.7%) was lower than the rates reported in open-access online survey research, and the difference between the control and intervention cohorts was not statistically significant (15/122, 12.3% vs 35/451, 7.8%; *P*=.12). Fewer surveys in the intervention cohort were flagged for a blank recruitment source (16/451, 3.5% vs 15/122, 12.3%; *P*<.001) and completion of multiple screeners (29/451, 6.4% vs 16/122, 13.1%; *P*=.02). The frequency of duplicated phone numbers was higher in the intervention survey (82/451, 18.2% vs 3/122, 2.5%; *P*<.001), although this difference was not statistically significant when restricted to individuals who provided a phone number (82/451, 18.2% vs 3/46, 6.5%; *P*=.06). Sensitivity analyses showed consistent patterns across alternative thresholds, and subgroup analyses did not show overall differences in fraud rates based on age, sex, or recruitment location.

**Conclusions:**

Enhanced fraud-deterrence procedures did not statistically significantly reduce overall fraud prevalence in a survey setting using clinic-linked, QR-based recruitment with modest incentives. A transparent scoring system provides a replicable approach for assessing survey integrity and may be preferable to reliance on eligibility gating alone.

## Introduction

Digital surveys allow timely data collection, reduce administrative burden, and facilitate participant engagement [1]. However, fraudulent responses, either from automated programs, intentional self-misrepresentation, or repeated submissions to obtain multiple incentives, can compromise data integrity and bias findings [2-4]. Online survey fraud is well documented, with multiple studies reporting over half of responses ultimately being excluded from analysis [5-8]. Additionally, the prevalence of online survey fraud is increasing, as evidenced by over three-quarters of responses before 2019 being usable compared with less than one-third since 2020 [9].

The existing literature describes a variety of prevention and detection strategies, including eligibility gating, CAPTCHA verification, device and metadata checks, and postsurvey logic assessments [5,10,11]. Because no individual indicator serves as a definitive marker, many studies rely on composite scoring methods across multiple heuristic signals to identify potential fraud [5,9]. However, most work focuses on open-access online recruitment (eg, social media and crowdsourcing platforms), where bot-generated or anonymous entries are common and increasing in complexity [12-17]. Fraud evolves quickly, and context-specific detection and prevention are emphasized but are rarely evaluated in real-world research settings, such as clinics where participants are recruited in person and subsequently complete surveys on their own devices [7,9,11,13,18].

Beyond detection, multiple layers of fraud deterrence, including recruitment control, content design, and postsubmission review, are recommended to improve data integrity [3,19]. Pre–data collection measures include single-use survey links, eligibility screening questions, and identity verification checks [9,20]. However, the relative contribution of these measures is poorly understood, and each comes with costs such as staff time, technological investment, or reduced response rates. It is therefore useful to assess both the prevalence of fraud and the effectiveness of deterrence strategies, particularly in pragmatic clinical research, where surveys may be embedded within established workflows. This evidence could inform whether fraud-detection investments for open-access surveys are proportionate to the associated risk in clinic-linked contexts.

This study evaluates indicator-based fraud signals across 2 iterations of a clinic-linked, moderately incentivized, postprocedure survey. A midstudy protocol modification created a natural experiment, allowing direct comparison of fraud indicators before and after the implementation of revised procedures. The aim of the study was to characterize indicator prevalence before and after workflow changes, with the goals of informing the calibration of fraud-detecting frameworks and assessing the impact of intentional measures to enhance digital survey integrity in a clinic-based setting.

## Methods

### Survey Context and Recruitment

This study was conducted as part of the larger ADOPT (Alternatives to Dental Opioid Prescribing After Tooth Extraction) clinical trial (NCT06275191), the details of which have been published previously [21]. As part of the trial, individual patients aged 12 to 25 years who underwent tooth extraction at 1 of 8 participating clinical practices in Kentucky and Indiana were eligible to complete a survey related to pain control, analgesic use, and overall satisfaction with their tooth extraction. The study survey was completed between postprocedure days 6 and 10. Within the study, participants aged 12 to 17 years were considered adolescents, and those aged 18 to 25 years were considered young adults; additionally, clinic locations affiliated with an academic medical center were considered “academic” clinics, and those not affiliated were considered “community” clinics.

This study is reported in accordance with the CHERRIES (Checklist for Reporting Results of Internet E-Surveys) [22] guidelines (Checklist 1) for web-based survey research to the extent possible for a clinic-linked recruitment workflow.

### Study Design

#### Survey Processes and Project Structure

**Figure 1.**
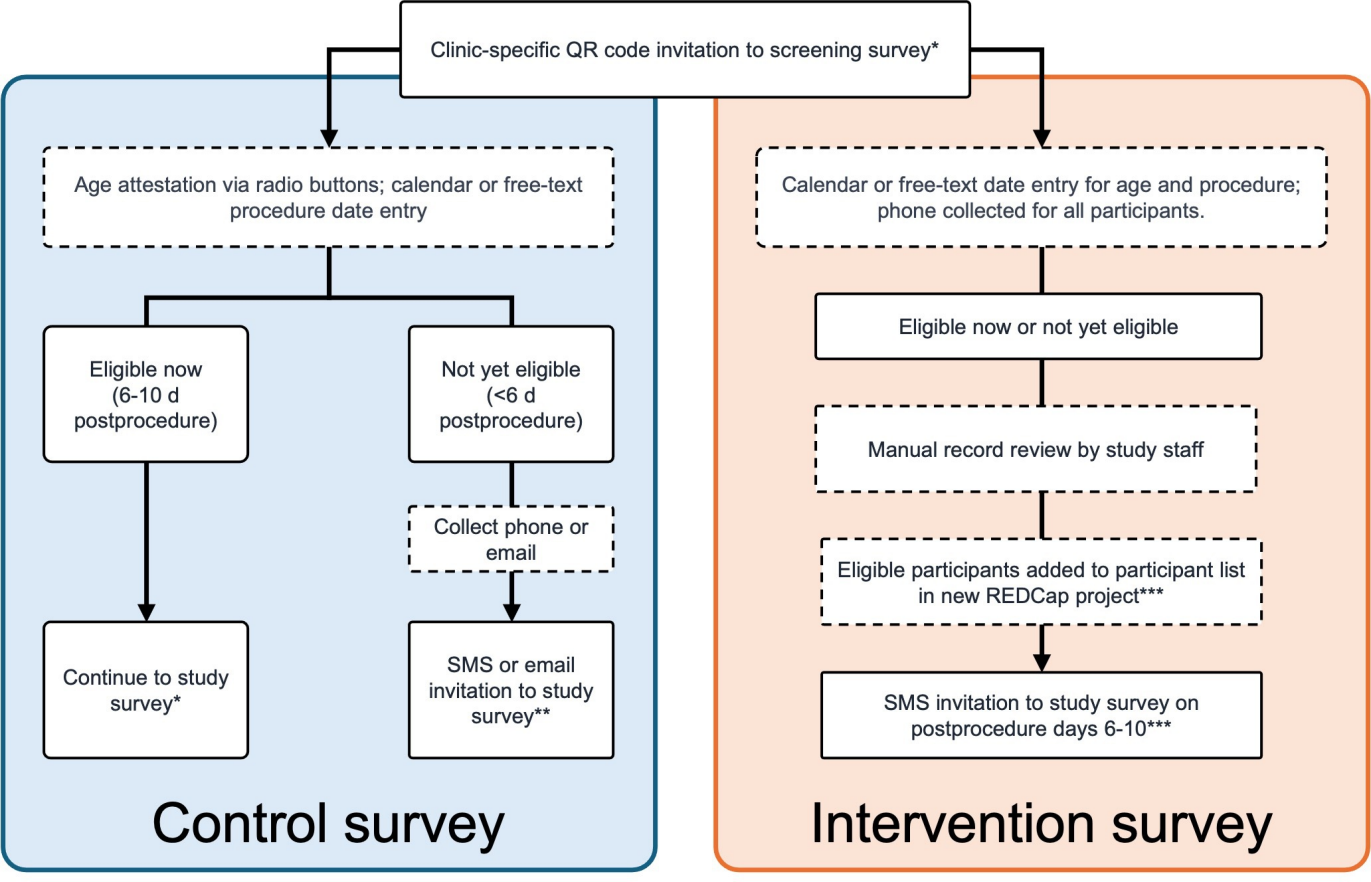
Survey recruitment workflows for the control (April 15-July 17, 2024) and intervention (July 18, 2024-August 15, 2025) iterations of the Alternatives to Dental Opioid Prescribing After Tooth Extraction (ADOPT; NCT06275191) survey. Both configurations began with a clinic-specific QR code linking to a screening survey. In the control survey, eligible participants proceeded directly to the study survey, whereas participants who were not yet eligible were recontacted on postprocedure day 6 via SMS or email. In the intervention survey, all screened participants provided their phone numbers and received an SMS invitation between postprocedure days 6 and 10 via a participant list. *Survey v1a REDCap project: same project as the screening survey; **Survey v1b REDCap project: invitation sent via public link; ***Survey v2 REDCap project: invitation sent via participant list.

The survey process and structure are outlined in Figure 1. Beginning in April 2024, recruitment flyers and invitation cards were distributed to all clinics participating in ADOPT. Each flyer and card contained a clinic-specific QR code that led individuals to a screening survey (ie, the QR code directed individuals to a single screening survey using a URL query parameter to automatically populate the clinic location in a hidden, noneditable field). At each clinic, an existing clinic staff member was designated to lead efforts of notifying potentially eligible participants about the survey. All survey data were collected in REDCap (Vanderbilt University), a secure, web-based platform hosted by the University of Kentucky [23].

After approximately 4 months of data collection, the research team identified vulnerabilities in the initial screening workflow, such as concerns about date manipulation and incorrect attestation of age. However, because the survey was embedded within established clinic workflows, modifications could not involve replacing QR codes or altering how clinics introduced the survey. Accordingly, fraud-deterrence revisions were implemented entirely within the backend REDCap infrastructure, allowing the research team to enhance security while minimizing the operational impact on clinic staff and preserving the patient-facing recruitment process.

#### Control Survey

The initial version of the screening survey used radio-button (eg, Are you between 12 and 25 y old?) and free-text (eg, When did you have your procedure?) questions to classify eligibility into 1 of 3 categories: eligible, not yet eligible, and ineligible. Eligible individuals were those between 12 and 25 years with a procedure date between 6 and 10 days ago; these participants could immediately proceed to the study survey (v1a), which was nested within the same REDCap project as the screening survey. Not yet eligible individuals were between 12 and 25 years but had a procedure less than 6 days ago (including future-dated procedures); these individuals’ preferred method of contact (email or phone number) was collected and used to send an invitation to a separate project with an identical version of the study survey (v1b) on postprocedure day 6. Ineligible individuals were informed why they were not eligible to complete the survey.

#### Intervention Survey

On July 18, 2024, the screening survey was modified to remove radio buttons and require free-text date of birth entry. The eligibility classification kept the same 3 categories (eligible, not yet eligible, and ineligible). However, all individuals who completed the screening survey were required to provide their phone number and were informed that, if eligible, they would receive an SMS text message invitation with a link to complete the survey. Screening records were manually reviewed by study staff, and nonduplicated records were populated into a second, identical copy of the study survey (v2) in a new REDCap project, using a participant list. To maintain recruitment workflows within participating clinics, the screening survey invitation QR code was not changed.

Once added to the v2 study survey participant list, eligible participants received individual SMS text message invitations between postprocedure days 6 and 10. Twilio cloud communications were used to send individualized invitations from REDCap to eligible participants.

Prior to beginning the v2 study survey, individuals were required to confirm, via free-text data entry with 1 attempt, their date of birth based on the value entered in the screening survey. A description of differences between the initial and modified surveys can be found in Table S1 in Multimedia Appendix 1.

### Analysis

Study surveys completed in either of the initial REDCap projects (v1a or v1b) were considered in the control cohort. Study surveys completed in project v2 were considered in the intervention cohort. Only surveys completed between April 15, 2024, and August 15, 2025, were included.

The evaluation included only completed study surveys matched to their corresponding screening surveys. Data from all 3 REDCap projects (v1a, v1b, and v2) were exported as .csv files using standardized REDCap reports. Records were merged across the files in Microsoft Excel using matched screening identifiers (clinic name, procedure date, and timestamp), with manual review to resolve ambiguous matches.

To screen for potentially fraudulent responses, a modified classification system, guided by previous publications [5,9,17,24], was used to assess the following indicators:

1. Completion time was calculated as end time minus start time [5,25,26]. The median completion time for all survey responses was 2 minutes 29 seconds (IQR 1 min 57 s to 3 min 55 s). Surveys with a completion time lower than the 5th or higher than the 95th percentile (ie, less than 1 min 19 s or more than 29 min 6 s) met this criterion.
2. Completion hour was identified as a screening or study survey completed before 6 AM or after 11 PM. Time-of-day flags have been used as supporting indicators with varying cutoffs [5,9]. We selected a more conservative 11 PM to 6 AM window to reflect reasonable waking hours for an adolescent and young adult population.
3. Multiple screeners were identified as multiple screening attempts from the same participating clinic in the hour preceding a successful screening attempt or multiple surveys only able to be linked to a single screening attempt [27]. These were included to identify individuals who may alter answers in a screening attempt to gain eligibility or those who may attempt the survey multiple times from the same successful link.
4. Duplicate phone number was defined as a phone number repeated across records in the study’s survey [9].
5. Blank recruitment location was defined as no recruitment location being captured by QR code in the screening survey [9]. This was included as a measure of nonstandard survey access.
6. Illogical responses were adapted from existing literature regarding illogical or internally inconsistent responses [5,28-30] and defined as individuals scoring 3 or more total points based on the sum of the below items met this criterion:
  - Inconsistent pain ranking (eg, average pain reported as 9/10, but worst pain reported as 3/10): 3 points
  - Selection of more than 50% (>3 of 7) of the possible races: 3 points
  - Opposing adverse reactions selected (eg, constipation and diarrhea): 2 points
  - Selection of more than 50% (>4 of 8) of possible adverse reactions and a final satisfaction rating of “very happy”: 2 points
  - Selection of “I don’t remember” for all questions regarding materials provided by the clinic: 1 point
  - Indicated taking 2 or more medications with the same mechanism (eg, naproxen and ibuprofen): 1 point

Indicator coding was performed in Excel using a combination of formulas, conditional logic, and manual inspection to generate the analytic dataset. Additionally, 2 auxiliary checks were conducted but did not contribute to scoring. First, an “impossible logic” trap question programmed only to display when prior responses were logically inconsistent (eg, when a participant selected both “yes” and “no” on a single radio item), intended to identify automated fraud, was assessed for completion. Second, email addresses, when available, were assessed for several potential indicators, including (1) nonsensical combinations of letters not typically used in email handles, (2) handle length greater than 22 characters or containing more than 6 numerical digits, (3) disposable or temporary email services, (4) duplication across survey responses, and (5) domains atypical for the target population [9,31]. However, email addresses were collected only from control cohort participants who screened prior to the eligibility window and elected not to provide a phone number (n=27). Neither impossible logic nor unusual email was flagged in any record.

The primary end point was the proportion of surveys classified as potentially fraudulent, defined a priori as 2 or more numbered indicators. This threshold was selected to reduce false-positive classification arising from any single indicator that could be attributed to benign causes (eg, slow connection increasing completion time, shared family phone numbers, etc), while still capturing surveys with patterns suggestive of intentional misrepresentation. Sensitivity analyses were conducted to evaluate the impact of less strict (1 or more indicators) and stricter (3 or more indicators) thresholds. Differences were assessed using 2-sided *t* test for continuous variables and chi-square or Fisher exact tests for categorical variables; due to small cell counts, race or ethnicity was compared using Fisher exact test with Monte Carlo simulation (10,000 replicates). Secondary analyses examined indicator prevalence across subgroups of age (adolescent vs young adult), sex, and recruitment location. Co-occurrence of indicator pairs was tabulated descriptively. Because phone number was only required in the intervention cohort, secondary analyses also assessed the effect of restricting duplicated phone number comparisons to individuals in the control cohort who provided a phone number. Statistical significance was assessed at ⍺=.05; all tests were 2-sided; and all statistical analyses were conducted in R (version 4.6.0; R Foundation for Statistical Computing; April 24, 2026).

### Ethical Considerations

This study and all procedural modifications to the survey were reviewed and approved by the University of Kentucky Institutional Review Board (protocol 80758). A waiver of documentation of informed consent was approved by the institutional review board; all participants reviewed a cover letter and agreed to participation before proceeding to the survey. A waiver of parental permission was also approved for the participation of minors (ages 12-17 years).

Identifiers collected during recruitment (ie, date of birth, phone number, and email) were used only for participant verification and reminder communications as part of the underlying clinical trial. For the analytic dataset, duplicated phone numbers and unusual emails were represented as binary indicators rather than the underlying values, and no other identifiers were retained. Age, sex, and race or ethnicity were retained at the record level. Upon completion of the study survey, individuals were directed to a payment form for the issuance of a US $20 Amazon gift card.

## Results

In total, 573 responses were included between the control (n=122) and intervention (n=451) cohorts. Individuals who completed the intervention survey were younger (mean 18.7, SD 3.1 vs mean 19.8, SD 3.2 y; *P*=.001), and more individuals who completed the intervention survey underwent tooth extraction in a community setting (368/451, 84.8% vs 75/122, 67.6%; *P*<.001). Full demographics are provided in Table 1.

**Table 1.** Participant demographics by cohorts (n=122 in the control cohort and n=451 in the intervention cohort)^a^.

Characteristics	Control cohort	Intervention cohort	*P* value
Age (y)^b^, mean (SD)	19.8 (3.2)	18.7 (3.1)	.001
Age group^b^, n (%)			<.001
Adolescent (12-17 y)	28 (23.9)	209 (46.3)	
Young adult (18-25 y)	89 (76.1)	242 (53.7)	
Sex^c^, n (%)			.70
Female	73 (62.9)	272 (60.7)	
Male	43 (37.1)	174 (38.8)	
Other	0 (0)	2 (0.4)	
Race or ethnicity^d^, n (%)			.05
American Indian or Alaska Native	0 (0)	1 (0.2)	
Asian	2 (1.8)	10 (2.2)	
Black or African American	14 (12.5)	37 (8.2)	
Hispanic or Latino	5 (4.5)	14 (3.1)	
White	83 (74.1)	351 (78.2)	
Multiple	3 (2.7)	32 (7.1)	
Other	5 (4.5)	4 (0.9)	
Clinic site^e^, n (%)			<.001
Academic	36 (32.4)	66 (15.2)	
Community	75 (67.6)	368 (84.8)	

a Demographic characteristics of participants completing a postprocedure pain survey within the ADOPT (Alternatives to Dental Opioid Prescribing After Tooth Extraction) clinical trial (NCT06275191). Participants were recruited from 8 dental practices in Kentucky and Indiana between April 15 and July 17, 2024 (control) and July 18, 2024, and August 15, 2025 (intervention).

b Data were missing for 5 (4.1%) participants in the control cohort.

c Data were missing for 6 (4.9%) and 3 (0.7%) participants in the control and intervention cohorts, respectively. Due to small sample size, sex categories were compared as binary variables, with the “Other” category (n=2) dropped from the statistical comparison.

d Data were missing for 10 (8.2%) and 2 (0.4%) participants in the control and intervention cohorts, respectively.

e Data were missing for 11 (9.0%) and 17 (3.8%) participants in the control and intervention cohorts, respectively.

During the study period, 2268 screening records were initiated (352 in the control period and 1916 in the intervention period), corresponding to 114 and 148 screening records per month, respectively. The screening-to-completion yield was lower in the intervention cohort than in the control cohort (451/1916, 23.5% vs 122/352, 34.7%), reflecting the additional filtering and verification steps incorporated into the intervention workflow.

Overall, 8.7% (50/573) of responses met the threshold for being potentially fraudulent while the rate was lower in the intervention cohort; this difference was not statistically significant (35/451, 7.8% vs 15/122, 12.3%; *P*=.12). Fewer surveys in the intervention cohort did not list a recruitment source (16/451, 3.5% vs 15/122, 12.3%; *P*<.001), and fewer attempted the screening survey multiple times (29/451, 6.4% vs 16/122, 13.1%; *P*=.02). Although duplicated phone numbers were more common in the intervention cohort (82/451, 18.2% vs 3/122, 2.5%; *P*<.001), the difference was not statistically significant when restricted to the 46 individuals who provided a phone number in the control survey (82/451, 18.2% vs 3/46, 6.5%; *P*=.06). The prevalence of individual indicators is displayed in Table 2.

**Table 2.** Potential fraud indicator prevalence by survey version (N=573)^a^.

Indicator	Control cohort (n=122), n (%)	Intervention cohort (n=451), n (%)	*P* value
Completion time (outside 5th-95th percentile)	16 (13.1)	42 (9.3)	.22
Completion hour (outside window)	6 (4.9)	20 (4.4)	.82
Multiple screeners	16 (13.1)	29 (6.4)	.02
Duplicated phone number	3 (2.5)	82 (18.2)	Cohort: <.001; providers: .06^b^
Blank recruitment source	15 (12.3)	16 (3.5)	<.001
Illogical responses	12 (9.8)	33 (7.3)	.36

a Frequency of potential fraud indicators among participants completing a postprocedure pain survey within the ADOPT (Alternatives to Dental Opioid Prescribing After Tooth Extraction) clinical trial (NCT06275191). Participants were recruited from 8 dental practices in Kentucky and Indiana between April 15 and July 17, 2024 (control) and July 18, 2024, and August 15, 2025 (intervention).

b Among control participants who provided a phone number (n=46), the rate was 6.5% (3/46), which was not statistically significantly different from the intervention rate (Fisher exact, *P*=.06).

Sensitivity analyses using alternate thresholds to flag potentially fraudulent responses are reported in Table 3. At a more permissive threshold of 1 or more indicators, 40.8% (234/573) of responses were flagged. At the strictest threshold of 3 or more indicators, less than 1% of surveys across the entire sample (2 in the intervention cohort and 3 in the control cohort) were flagged. Only 1 survey in the control cohort triggered more than 3 indicators.

**Table 3.** Sensitivity analyses of potentially fraudulent response classification at alternate indicator thresholds^a^.

Potentially fraudulent response threshold score	Control cohort (n=122), n (%)	Intervention cohort (n=451), n (%)	Total (N=573), n (%)	*P* value^b^
≥1 indicator	49 (40.2)	185 (41.0)	234 (40.8)	.86
≥2 indicators (primary)	15 (12.3)	35 (7.8)	50 (8.7)	.12
≥3 indicators	3 (2.5)	2 (0.4)	5 (0.9)	.07

a Postprocedure pain survey responses (N=573) were collected within the ADOPT (Alternatives to Dental Opioid Prescribing After Tooth Extraction) clinical trial (NCT06275191). Participants were recruited from 8 dental practices in Kentucky and Indiana between April 15 and July 17, 2024 (control) and July 18, 2024, and August 15, 2025 (intervention).

b Control vs intervention.

Subgroup analyses suggested no differences in the prevalence of potentially fraudulent survey responses between adolescents and young adults (15/237, 6.3% vs 30/331, 9.1%; *P*=.23), between females and males (26/345, 7.5% vs 19/217, 8.8%; *P*=.60), or between academic sites and community sites (13/102, 12.7% vs 34/443, 7.7%; *P*=.10). Individual indicator analyses suggested that more respondents who underwent their procedure in an academic setting were flagged for illogical responses (16/102, 15.7% vs 28/443, 6.3%; *P*=.002), and more females were flagged for completion hour (21/345, 6.1% vs 5/217, 2.3%; *P*=.04); otherwise, there were no statistically significant differences in subgroup analyses. Subgroup and individual indicator analyses are presented in Tables S2-S4 in Multimedia Appendix 1.

Finally, among the 50 surveys identified as potentially fraudulent, the most common co-occurring indicator pairs differed between cohorts. In the 15 surveys flagged in the control cohort, the most common indicator pairs were a blank recruitment source with multiple screeners (n=5), illogical responses with multiple screeners (n=5), and illogical responses with a short or long completion time (n=3). Across the 35 surveys flagged in the intervention cohort, a duplicated phone number most often occurred with completion hour (n=8), multiple screeners (n=7), illogical responses (n=6), and a short or long completion time (n=6; Table S5 in Multimedia Appendix 1).

## Discussion

### Principal Results and Comparison With Existing Literature

In this study evaluating the impact of implementing enhanced fraud-deterrence measures on indicators of survey integrity, the overall prevalence of potentially fraudulent responses (50/573, 8.7%) was lower than rates reported in open-access online surveys. Although the rate was numerically lower after implementation of additional security measures (35/451, 7.8% vs 15/122, 12.3%), this difference was not statistically significant. Sensitivity analyses using alternative thresholds for fraud detection yielded consistent results, suggesting that the observed findings were robust to different cutoff criteria.

Although the deterrence measures deployed in this study did not reduce the prevalence of potentially fraudulent responses, the overall rate was substantially lower than that reported in many online surveys [5,9,32]. Notably, the use of preselection criteria in this study, even in the control version (ie, distribution of physical cards at specific oral surgery clinics), aligns with lower potential fraud rates in invited compared with open online social media or crowdsourcing surveys [33]. The absence of flagged indicators in auxiliary checks (impossible logic and unusual email) suggests that automated or bot-like activity was limited in this study, potentially owing to the use of CAPTCHAs and intentional distribution methodologies.

The distribution of fraud indicators differed between cohorts in patterns consistent with the modifications made in the intervention survey. For example, both a blank recruitment source and multiple screening attempts were less common in the intervention cohort. This is consistent with not immediately informing individuals why they did not pass the screen in the intervention cohort. In the control cohort, individuals may have been prompted by the explicit ineligibility message to use the browser back button to revise their answers, which cleared the URL-embedded location parameter and generated multiple screening attempts. Additionally, duplicated phone numbers were more frequently flagged in the intervention cohort, but this difference was no longer statistically significant when analyses were restricted to participants who provided a phone number in both cohorts. This suggests that a repeated phone number may function as a more sensitive marker of potential fraud when a phone number is required, aligning with published evidence that phone number is among the highest-yield fraud indicators [9]. Additionally, co-occurrence patterns differed by cohort, where control responses often combined a blank recruitment source with multiple screening attempts or illogical responses, but intervention responses combined a repeated phone number (a required field) with other indicators. This suggests that different deterrence strategies may capture fundamentally different characteristics.

Subgroup analyses suggest that findings were largely consistent across populations. For example, while adolescents may be more likely to provide illogical or inconsistent responses (eg, due to misunderstanding vs fraud), the overall prevalence of potential fraud did not differ between adolescents and young adults, and none of the individual indicators were statistically significantly different between these groups (Table S2 in Multimedia Appendix 1). In contrast, illogical responses were more common in academic vs community sites (16/102, 15.7% vs 28/443, 6.3%, *P*=.002; Table S4 in Multimedia Appendix 1) despite no significant difference in overall flagging by site type. This suggests that some indicators may be sensitive to setting-level patient or workflow characteristics (eg, procedural complexity) rather than genuine fraud and that individual sites may need to calibrate indicators accordingly.

### Limitations

This study has several important limitations. First, although fraud indicators were informed by existing literature, some elements of the scoring system were investigator-defined and may not capture the full scope of deceptive behaviors. Additionally, although all indicators in this analysis contributed equally to the composite score, certain indicators may be more informative than others depending on the population and incentive structure. Some indicators may reflect circumstantial differences (eg, distracted responding, poor internet connection or page refreshes, or variations in sleep times) rather than intentional fraud and were therefore applied only within a composite scoring framework. Other indicators, such as a repeated phone number, may carry stronger evidence of fraud. Similarly, potential indicators such as IP address were inconsistently available in our institution’s specific REDCap instance. Second, our focus on suspected fraud does not include an assessment of response validity; for example, some logic-based indicators could have been triggered by participants who misunderstood survey items rather than intentionally or carelessly provided inconsistent responses, which is why these indicators contributed to a composite “illogical response” score. Third, the relatively small sample size, specifically of the control cohort, limits the power to detect differences between study versions, and differences in specific indicators may reflect design differences (eg, a required phone number) rather than behavior change. Similarly, cohort assignment was not randomized, resulting in meaningful demographic differences between groups. Although subgroup analyses were similar, residual confounding cannot be fully excluded. Our sensitivity analyses at alternative indicator thresholds also do not assess whether including or excluding flagged responses meaningfully changes the substantive findings of the parent ADOPT trial; at each threshold, the absence of a significant between-group difference indicates similar fraud signal levels in both cohorts, but that does not establish that the underlying study outcomes were unaffected by suspected fraud. Additionally, fraud screening was limited to indicators available in the survey, meaning that superficially plausible bot-generated data or participant re-enrollment from a new device could have been missed. We also did not have access to independent ground-truth fraud labels because of the blinding within the parent clinical trial. Without a gold standard, our indicators capture signals of potential fraud rather than confirmed fraud. Accordingly, this complicated potentially more useful analyses, such as the development of a weighted scoring system. Finally, because recruitment for this survey was clinic-linked, QR code–based, and modestly incentivized, findings may not be generalizable to online-only or higher-incentive contexts where fraud is more prevalent.

Several important lessons were learned from this study. First, eligibility gating alone does not prevent potentially fraudulent responses; individuals may still attempt re-entry or manipulation after passing an initial screening step. In our intervention design, the combination of a standardized screening survey with delayed eligibility notification and manual review appeared to deter certain forms of manipulation, even though these improvements were not captured by significant reductions in overall fraud prevalence. Second, although overt fraud was uncommon, nonfraudulent but clinically meaningful inconsistencies (eg, inconsistent pain ranking) still occurred, underscoring the need to complement fraud detection with strategies aimed at improving response quality. Overly restrictive fraud detection risks incorrectly classifying legitimate responses, resulting in data loss, particularly from individuals with low literacy, limited internet access, or shared devices [7]. Collectively, these lessons emphasize that data integrity in digital health surveys requires a layered approach involving recruitment design, content structure, and ongoing review, rather than reliance on a single safeguard. Finally, fraud mitigation and detection required significant investment from the study team to maintain operations within participating clinics. While initial survey invitations were designed to integrate seamlessly into existing clinic workflows, modifications needed to be implemented without placing undue burden on clinics. This required several backend security enhancements to minimize survey “downtime,” maintaining the initial QR codes distributed to clinics, and extensive manual oversight during the 10-day designed delay in the survey to ensure all individuals who scanned a QR code in the control condition had the opportunity to complete the survey between postprocedure days 6 and 10. Monthly survey volume did not meaningfully decline after implementation. The control period averaged approximately 40 responses per month, compared with 35 per month in the intervention, likely attributable to seasonal fluctuations, given the control period occurred during summer months when tooth extractions in adolescents and young adults are more frequent.

This study contributes to the emerging body of work on data integrity in digital health research by providing an evaluation of survey response quality within a technologically savvy, clinic-recruited population. By applying a defined, point-based fraud scoring system, we offer a transparent and replicable approach to evaluating potentially fraudulent responses in REDCap and similar survey platforms. These findings support the implementation of reasonable safeguards proportionate to the actual fraud risk in clinic-linked, incentivized surveys, considering the operational cost of integrity measures and recognizing that the fraud prevalence from open-link surveys may not directly translate to clinical settings.

Several opportunities exist to extend this work. The fraud-scoring system proposed in this study could be validated in additional clinical and public health settings, including those with higher incentives or different recruitment pathways. Additionally, future studies could examine whether specific fraud indicators are associated with meaningful differences in reported survey outcomes. Finally, more advanced analytic approaches, such as machine learning classifiers, anomaly detection, or latent profile analysis, could identify patterns not captured by rule-based scoring alone.

### Conclusions

The overall prevalence of potentially fraudulent responses in this clinic-linked, incentivized postoperative pain survey was lower than that reported in publicly available online surveys and was not statistically significantly reduced after the implementation of fraud-deterrence strategies. These findings suggest that both the patterns and the prevalence of fraud may differ from open-access online recruitment and support a context-sensitive, proportionate approach to fraud detection in clinical research.

## Supplementary material

10.2196/90159 Multimedia Appendix 1 Survey security procedures and descriptive analyses of fraud indicator prevalence and co-occurrence in the ADOPT (Alternatives to Dental Opioid Prescribing after Tooth Extraction) clinical trial (NCT06275191).

10.2196/90159 Checklist 1 CHERRIES checklist.

## Abbreviations

ADOPT: Alternatives to Dental Opioid Prescribing After Tooth Extraction
CHERRIES: Checklist for Reporting Results of Internet E-Surveys
