# Supplementary material for: Practical Fraud Detection and Prevention in Incentivized Online Surveys: Secondary Analysis of the ADOPT Study
Source: J Med Internet Res. 2026 Jul 31;28:e90159. doi: 10.2196/90159 (PMC13426555; doi:10.2196/90159)
Supplement: Multimedia Appendix 1 [file jmir-v28-e90159-s001.docx]

**Supplementary Materials**

The following supplementary materials provide additional context and results of descriptive analyses for postoperative pain survey responses collected in the ADOPT clinical trial (NCT06275191) at eight oral surgery practices throughout Kentucky and Indiana between April 15, 2024 and August 15, 2025.

Table S1. Initial and Modified Survey Security Procedures

Table S2. Fraud Indicator Prevalence by Age Group

Table S3. Fraud Indicator Prevalence by Sex

Table S4. Fraud Indicator Prevalence by Recruitment Location

Table S5. Co-occurrence of Fraud Indicators among Surveys Classified as Potentially Fraudulent

**Table S1. Initial and Modified Survey Security Procedures**

|  | Control Survey | Intervention Survey |
| --- | --- | --- |
| Active Dates | Apr. 15,2024 – Jul. 17, 2024 | Jul. 18, 2024 – Aug. 15, 2025 |
| CAPTCHA Use in Screening Survey | Yes | Yes |
| Age Screening | Radio button confirmation | Date of birth |
| Notification of Ineligibility | Yes | No |
| Notification of Reason for Ineligibility | Yes | No |
| Number of Attempts Allowed in Screening Survey | Unlimited | Unlimited |
| Access to Study Survey for Individuals Immediately Eligible | Yes, automatic continuation within REDCap | No |
| Required Fields in Screening Survey | Procedure date  Age attestation | Procedure date  Age (date of birth)  Phone number |
| Method of Notification for Delayed Survey | Email or SMS text message | SMS text message after manual review |
| Study Survey Security | Publicly available link | Individual one-time link via participant list |
| Age Verification in Study Survey | None | Date of birth, single attempt |

**Table S2. Fraud Indicator Prevalence by Age Group**

| Indicator | Adolescent,  12-17  (n=237) | Young Adult, 18-25 (n=331) | p value |
| --- | --- | --- | --- |
| Completion Time (outside 5^th^-95^th^ percentile) | 20 (8.4%) | 37 (11.2%) | .284 |
| Completion Hour (outside window) | 8 (3.4%) | 18 (5.4%) | .246 |
| Multiple Screeners | 16 (6.8%) | 24 (7.3%) | .818 |
| Duplicated Phone Number | 38 (16.0%) | 47 (14.2%) | .546 |
| Blank Recruitment Source | 11 (4.6%) | 15 (4.5%) | .951 |
| Illogical Responses | 16 (6.8%) | 28 (8.5%) | .453 |
| Potentially fraudulent (≥2 indicators) | 15 (6.3%) | 30 (9.1%) | .234 |

From survey responses with available age data (n=568; 5 control-cohort responses with missing age excluded). Chi square test used for all analyses.

**Table S3. Fraud Indicator Prevalence by Sex**

| Indicator | Female (n=345) | Male (n=217) | p value |
| --- | --- | --- | --- |
| Completion Time (outside 5^th^-95^th^ percentile) | 33 (9.6%) | 24 (11.1%) | .568 |
| Completion Hour (outside window) | 21 (6.1%) | 5 (2.3%) | .038 |
| Multiple Screeners | 24 (7.0%) | 15 (6.9%) | .984 |
| Duplicated Phone Number | 54 (15.7%) | 30 (13.8%) | .554 |
| Blank Recruitment Source | 11 (4.9%) | 9 (4.1%) | .668 |
| Illogical Responses | 25 (7.2%) | 19 (8.8%) | .517 |
| Potentially fraudulent (≥2 indicators) | 26 (7.5%) | 19 (8.8%) | .604 |

From survey responses with available sex reported as Female or Male (n=562; 2 responses with sex reported as “Other” and 9 with missing sex excluded). Chi square test used for all analyses.

**Table S4. Fraud Indicator Prevalence by Recruitment Location**

| Indicator | Academic (n=102) | Community (n=443) | p value |
| --- | --- | --- | --- |
| Completion Time (outside 5^th^-95^th^ percentile) | 11 (10.8%) | 45 (10.2%) | .851 |
| Completion Hour (outside window) | 3 (2.9%) | 22 (5.0%) | .598* |
| Multiple Screeners | 13 (12.7%) | 32 (7.2%) | .068 |
| Duplicated Phone Number | 14 (13.7%) | 69 (15.6%) | .639 |
| Blank Recruitment Source^†^ | -- | -- | -- |
| Illogical Responses | 16 (15.7%) | 28 (6.3%) | .002 |
| Potentially fraudulent (≥2 indicators) | 13 (12.7%) | 34 (7.7%) | .100 |

From survey responses with available site information (n=545). Academic sites were those affiliated with an academic medical center; all others were community sites. Unless otherwise specified, Chi square test used for all analyses.

*Fisher’s exact test used.

†Not applicable for this subgroup analysis based on recruitment location.

**Table S5. Co-occurrence of Fraud Indicators among Surveys Classified as Potentially Fraudulent**

| **Indicator Pair** | **Overall (n=50)** | **Control (n=15)** | **Intervention (n=35)** |
| --- | --- | --- | --- |
| Completion Hour + Repeated Phone Number | 10 | 2 | 8 |
| Multiple Screeners + Repeated Phone Number | 8 | 1 | 7 |
| Completion Time + Repeated Phone Number | 7 | 1 | 6 |
| Repeated Phone Number + Illogical Responses | 7 | 1 | 6 |
| Completion Time + Illogical Responses | 6 | 3 | 3 |
| Multiple Screeners + Blank Recruitment Source | 5 | 5 | 0 |
| Multiple Screeners + Illogical Responses | 5 | 4 | 1 |
| Completion Time + Multiple Screeners | 4 | 2 | 2 |
| Completion Time + Blank Recruitment Source | 3 | 3 | 0 |
| Completion Hour + Multiple Screeners | 2 | 0 | 2 |
| Completion Time + Completion Hour | 2 | 1 | 1 |
| Completion Hour + Blank Recruitment Source | 1 | 0 | 1 |
| Completion Hour + Illogical Responses | 1 | 0 | 1 |
| Repeated Phone Number + Blank Recruitment Source | 1 | 0 | 1 |
| Blank Recruitment Source + Illogical Responses | 1 | 1 | 0 |

Pairs of fraud indicators triggered together from the n=50 surveys classified as potentially fraudulent (≥2 indicators). Pairs are sorted by overall frequency. Surveys with more than 2 indicators contribute to multiple pairs (eg, a survey with 3 indicators contributes to 3 pair combinations).
