# Supplementary material for: Practical Fraud Detection and Prevention in Incentivized Online Surveys: Secondary Analysis of the ADOPT Study
Source: J Med Internet Res. 2026 Jul 31;28:e90159. doi: 10.2196/90159 (PMC13426555; doi:10.2196/90159)
Supplement: Checklist 1 [file jmir-v28-e90159-s002.docx]

**CHERRIES Checklist**

This checklist accompanies the manuscript entitled "Practical Fraud Detection and Prevention in Incentivized Online Surveys: Lessons from the ADOPT Study," reporting results from postoperative pain survey responses collected in the ADOPT clinical trial (NCT06275191) at eight participating dental practices in Kentucky and Indiana between April 15, 2024 and August 15, 2025. The checklist follows the CHERRIES guidelines (Eysenbach 2004) for reporting web-based survey research, adapted where appropriate to the clinic-linked recruitment workflow used in this study.

| ***Item Category*** | ***Checklist Item*** | ***Description*** |
| --- | --- | --- |
| Design | Describe survey design | See manuscript text. Population was recruited as individuals age 12-25 undergoing tooth extraction in clinics participating in the ADOPT study. |
| IRB approval and informed consent | IRB approval | University of Kentucky IRB 80758. Individual modification requests approved before each survey change. |
|  | Informed consent | Cover letter in lieu of full informed consent |
|  | Data protection | All data securely stored in University of Kentucky REDCap instance. |
| Development and pre-testing | Development and testing | Initial survey piloted with University of Kentucky patients (n=8) prior to launch. |
| Recruitment process | Open survey vs. closed | Clinic-linked recruitment (see manuscript text) |
|  | Contact mode | Clinic-linked recruitment (see manuscript text) |
|  | Advertising the survey | QR-coded flyers and pocket cards available in clinics participating in ADOPT study (see manuscript text) |
| Survey administration | Web/E-mail | Survey hosted on REDCap. Final survey invitation varied based on control vs. intervention cohort. |
|  | Context | Survey hosted on REDCap using clinic-linked recruitment. |
|  | Mandatory/voluntary | Voluntary |
|  | Incentives | $20 Amazon gift card on completion of the final survey |
|  | Time/date | April 2024-August 2025 |
|  | Randomization | No items were randomized or alternated |
|  | Adaptive questioning | Branching logic/adaptive questioning was used throughout the survey |
|  | Number of items | The survey varied based on adaptive questioning but was approximately 20 questions. |
|  | Number of screens (pages) | The final survey was administered over 4 pages. |
|  | Completeness check | Responses were optional (vs. required with a “refuse to answer” option) to align with established instruments (e.g., PROMIS pain interference matrices). |
|  | Review step | Backward navigation was not allowed. |
| Response Rates | Unique site visitor | N/A |
|  | View rate | N/A |
|  | Participation rate | N/A |
|  | Completion rate | N/A |
| Preventing multiple entries | Cookies used | Not supported |
|  | IP check | Not supported |
|  | Log file analysis | Several methodologies outlined in manuscript, varying by control vs. intervention cohort (e.g., date of birth match) |
|  | Registration | Intervention cohort required manual phone number entry and text invitation. |
| Analysis | Incomplete responses | N/A. Study focused on fraud indicators, not survey content. |
|  | Atypical timestamp | See methods. Included as fraud indicator. |
|  | Statistical correction | N/A. Representativeness was not an analytic objective of this study. |
